# Effect of mindfulness meditation protocol in subjects with various psychometric characteristics at high altitude

**DOI:** 10.1002/brb3.1604

**Published:** 2020-03-23

**Authors:** Disha Bhanushali, Rahul Tyagi, Nitin Limaye (Rishi Nityapragya), Akshay Anand

**Affiliations:** ^1^ Ved Vignan Maha Vidya Peeth Sri Sri Institute of Advanced Research Bangaluru India; ^2^ Neuroscience Research Lab Department of Neurology Postgraduate Institute of Medical Education and Research Chandigarh India

**Keywords:** advance meditation, High altitude, pO2, Prakriti, SKY

## Abstract

**Introduction:**

Incidence of high altitude‐related sickness is increasing due to more number of people visiting the areas of high altitude which may result in life‐threatening conditions including acute mountain sickness (AMS), high altitude pulmonary edema (HAPE), high altitude cerebral edema (HACE), and High‐altitude pulmonary hypertension (HAPH). We hypothesized that an advanced yoga regimen may be beneficial in dealing with the physiology of acclimatization.

**Methods:**

Anthropometric, Biochemical, and Psychological assessments were carried out in 48 participants before and after the advance meditation program (AMP) in the experimental group. Individuals with an age range of 20–65 years with no comorbidities were included in the study. Participants were exposed to AMP for 4 days. All assessments were carried out at the baseline and after the course. Prakriti was constituted for all participants using a standard questionnaire. The study was carried out after obtaining the written informed consent as per the guidelines outlined by the Institute Ethics Committee.

**Results:**

Po2 and glucose levels were found significantly reduced along with changes in the Happiness index, anxiety, and mental well‐being. However, participants with lowered Po2, after 4 days of mindfulness intervention, showed a positive outcome measured by the established scales of anxiety, happiness, and information processing. Psychometric or *Prakriti* wise analysis revealed that subject with “*Pitta*” constitution exposed to high altitude and advance meditation showed changes in more parameters than “*Vatta*” or “*Kapha*” Constitution.

**Conclusions:**

Advance meditation in the high altitude zone confers biochemical and neuro‐cognitive benefits. Molecular studies may require to understand the role of hypoxic condition in improving the disease state.

## Graphical abstract

This article provides provocative data that makes a compelling argument for the association between the practice of meditation at high altitude and biochemical and psychological parameters according to the specific personality traits. It was also noted that individuals with persistent low sPo2 had significant improvement in psychological parameters. The data may be used for future studies dealing with sickness associated with high altitude and treatment strategies at high altitude.

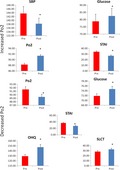

## 1 INTRODUCTION

High altitude sickness is a growing concern due to the increased number of travelers, sportspersons, adventurers, pilgrims, army personnel, and even non‐resident locals living at high altitude >2,500 m (Kapoor, Narula, & Anand, 2004; Paralikar & Paralikar, 2010). Mild to life‐threatening clinical conditions such as acute mountain sickness (AMS), high altitude pulmonary edema (HAPE), high altitude cerebral edema (HACE), and High‐altitude pulmonary hypertension (HAPH), have been reported (Kapoor et al., 2004) at high altitude. The physiological effects of high altitude begin at 1,500–3,500 m (m) and gradually increase at 3,500–5,500 m and become severe at extremely high altitude (above 5,500 m) (Paralikar & Paralikar, 2010). Hypoxic physiological conditions develop mainly due to reduced atmospheric pressure resulting in the corresponding reduction in the partial pressure of oxygen (PO2). Adaptation to the hypoxic HA conditions requires synergic functioning of respiratory, cardiac, and hematological system of the human body in order to enhance the bio‐availability of oxygen at the cellular levels. Gradual acclimatization by traveling through road has remained a preventive tool to adapt to the conditions which takes 3–4 days. Drugs like acetazolamide, dexamethasone, nifedipine are recommended for early acclimatization. Symptomatic therapies include aspirin, ibuprofen for headache and promethazine for nausea. The mechanism of action of these drugs shows that it may affect kidney, RBCs, Lungs, and brain and may contraindicate the existing pathophysiology of individuals (Ono, Morifusa, Ikeda, Kunishige, & Tohma, 2017; Paralikar & Paralikar, 2010).

In the absence of failure to rescue high altitude‐related complications, Yoga interventions have been suggested. The rationale for its application is based on anecdotes of sages living in high altitudes and surviving for long despite reduced food intake and oxygen. Whether Yoga can be effectively characterized to systematically apply it for a preventive role in acclimatization‐related complications, either as pre‐conditioning module or as onsite intervention program needs comprehensive research and evaluation. A case‐control study conducted in 12 Caucasian trainees and 12 sea‐level residents at 5,050 m altitude showed improved oxygen transport, minimal increase in ventilation, and improved hematological changes (Bernardi et al., 2007). Similarly, Yogic breathing was found to be helpful in maintaining oxygenation in the hypobaric chambers equivalent to 5,000 m altitude (Bernardi et al., 2001). In a study conducted on 200 fully acclimatized Indian soldiers at a high altitude of Leh (India), yogic practices were found to be superior to the physical exercises in improving the physiological, biochemical and psychological functions. However, the role of *Prakriti* or individualized personality type constitution in the efficiency of acclimatization has not yet been studied (Himashree, Mohan, & Singh, 2016). According to the ancient system of Indian medicine, that is, Ayurveda, individual variability may be classified phenotypically into seven broad constitution types termed Prakriti, among which Vata (V), Pitta (P), and Kapha (K), acts as the most contrasting constitutions (Aggarwal et al., 2010). These phenotypes respond differently to diet, nutrition, medications, drugs, and environmental stimuli. Predisposition to specific diseases may require a balance between these prakritis. Rotti et al reported 80% concordance between Prakriti and software‐based prediction models(Rotti et al., 2014). Altitude may affect or improve different individuals based on their prakriti constitution.

A breath‐based mindfulness meditation sequence such as the Art of Living (AOL) Sudarshan Kriya Yoga (SKY) founded by Sri Sri Ravi Shankar has been shown to reduce systolic blood pressure, diastolic blood pressure and respiration rate in subjects besides development of self‐awareness and harmonization of the mind with the body. This could be useful for the enhancement of human performance and happiness, necessary to rescue harsh and hypoxic conditions. Regardless of various published evidence, the mechanisms by which yogic breathing positively impacts the individual's compensatory reserves are not yet completely understood (Somwanshi, HSM, & Kolpe, 2013). Studies have reported the beneficial impact of SKY on lowering anxiety, depression, and stress as compared to controls (Chandra, Jaiswal, Singh, Jha, & Mittal, 2017; Kjellgren, Bood, Axelsson, Norlander, & Saatcioglu, 2007) but none of these studies have been systematically evaluated in harsh conditions or high altitude. In the current study, we analyzed the effect of SKY and related meditation techniques (or mindfulness meditation, herein called SKY) among individuals seeking to adapt to the hypoxic environment, caused due to abrupt ascent to high altitude, by flight, based on their *Prakriti* constitution.

## 2 METHODOLOGY

### 2.1 Recruitment and randomization of participants

A total of 48 subjects were recruited for the study. The study was approved by the Institutional Ethics Committee of SSIAR, Bangaluru, India (vide SSIAR/IEC/05) and PGIMER, Chandigarh, India (PGI/IEC/2019/000643). Special regulatory permission was granted by the director, SSIAR, Bangaluru, India. These subjects were enrolled in the Art of living (AOL) Advanced Meditation Program in Mahabodhi Meditation Center, Leh, India. The study was carried out after obtaining the written informed consent as per the guidelines outlined by the Institute Ethics Committee. Randomization was not possible since recruitment was available to a limited number of program participants. The current study was, therefore, a single‐arm exploratory study.

### 2.2 Inclusion and exclusion criteria

Individuals with age range of 20–65 years with no comorbidities were included in the study after obtaining their written informed consent. Subjects were exposed to SKY in order to perform the advanced level meditation. Inclusion and exclusion criteria are tabulated in Table 1.

**Table 1:** Inclusion and exclusion criteria

S. No.	Variables units	Inclusion criteria	Exclusion criteria
1	Gender	Male, Female and Third Gender	NA
2	Age Range (in years)	20–65	<20 or >65
3	Education	Literate	Illiterate
3	General Health	No co‐morbidities*	NA
4	Consent	Yes	NA
5	Individuals who have prior exposure to SKY	Yes	NA

^*^Comorbidities: Heart Diseases, Cancer, Chronic lung disease, Chronic liver disease, Chronic lower back pain, Chronic neurological diseases, Moderate or severe kidney disease, Any other chronic illness, Any major Surgery in the past.

### 2.3 Sudarshan Kriya Yoga (SKY) and related meditation techniques protocol

The detailed protocol was administered under the strict supervision of authorized teachers Rishi Nityapragya (Nitin Limaye) and Dr. Disha Bhanushali. The protocol included Yoga exercises, Pranayama, SKY, Padmasadhana, Nadi shodhan/anulom‐vilom pranayama, Guided Meditations, Practice of silence, practical wisdom, spiritual music and selfless services. Duration: Entire protocol was carried out for the duration of 4 days. The course started in the early morning (5 a.m.) till late evenings (8 p.m.). No other medication was allowed to the participants, unless medical emergencies. All of the enrolled participants attended all sessions.

Various studies have used SKY for clinical efficacies (Brown & Gerbarg, 2005; Mathersul et al., 2019); however, there are less studies describing the effect of advanced meditation.

The detailed descriptions of the SKY breathing technique and Advanced Meditation Program are as follows:

1. Yoga exercises, Pranayama, Sudarshan Kriya Yoga

*Suryanamaskar (Sun salutation)* along with a set of other supine, sitting and standing *asanas* were performed under a Yoga expert.

A protocol called *Padmasadhana* was done. Which included the below:

A. Set of 18 Yogasanas

- Body rotation
- Locust pose with right leg
- Locust pose with left leg
- Locust pose
- Cobra pose
- Superman
- Bow pose
- Crocodile pose
- Boat pose
- Relieving posture right leg
- Relieving posture Left leg
- Relieving posture
- Shoulder stand
- Dancing Shiva pose with right leg
- Dancing Shiva pose with left leg
- Half Spinal twist with right leg
- Half Spinal twist with left leg
- Mountain pose

- 9 rounds of Nadi shodhan/anulom‐vilam pranayam
- Guided meditation for 20 min
- 9 rounds of Nadi shodhan/anulom‐vilom pranayama

This was followed by *3 stage Pranayama,* Bhastrika, and *Sudarshan Kriya Yoga*.

3 stage *Pranayama:* In every stage, the position of the hands is different. Hands are kept on the pelvic bone in the first stage, on chest area in the second stage, and on the back of the shoulder in the third stage. All stages involve the same procedure of breathing, that is, breath in *Ujjai* for a definite period, to hold the breath for a definite period and then to breathe out *Ujjai *for a definite period (Zope & Zope, 2013). This involves experiencing the conscious sensation of the breath touching the throat. This slow breath technique (6–8 breaths per minute) increases airway resistance during inspiration and expiration and controls airflow so that each phase of the breath cycle can be prolonged to an exact count. The subjective experience is physical and mental calmness with alertness. *Bhastrika* (Bellows Breath): In this technique, air is rapidly inhaled and forcefully exhaled at a rate of 20 breaths per minute. It involves up and down hand movements and is coordinated with synergetic fast breath in and out through the nostrils (Zope & Zope, 2013). It causes excitation followed by calmness. “*Om” *is chanted three times with very prolonged expiration and is followed by *Sudarshan Kriya (*Zope & Zope, 2013 *).*

1. *Sudarshan Kriya Yoga* (SKY): *Sudarshan Kriya* is a Sanskrit term meaning “internal visualisation by purifying technique” and is also known as healing breath technique. It is an advanced form of rhythmic, cyclical breathing with slow (20), medium (40), and fast (40) cycles performed three times. This *Kriya* ends with keeping the body immobile for 1 min and paying attention to the body followed by 5–8 long breaths and lying down in supine position for deep meditation with eyes closed for few minutes (Zope & Zope, 2013).
2. Specially designed guided meditations by Sri Sri Ravi Shankar: Guided meditation techniques which vastly emphasized on the cleansing of *chakras* and upliftment of energy to higher *chakras*.
3. Practice of silence, practical wisdom, spiritual music, and selfless services was incorporated in the remaining part of the protocol for the duration of 4 days (Table 2).

**Table 2:** SKY advance meditation protocol

S. No.	Protocol	Duration
1	LOOSENING PRACTICE	5 min (morning)
2	YOGASANAS (Yoga Postures) and PRANAYAMA	2 hr (morning)
2a	Surya Namaskar	
2b	Various stretches	
2c	Padmasadhana	
2c‐1	Set of 18 Asanas	
2c‐2	Anulom vilom Pranayama (alternate nostril breathing)	
2c‐3	Guided Meditation	
2c‐4	Anulom vilom Pranayama (alternate nostril breathing)	
3	3 stage Pranayam	
4	Bhastrika Pranayama (bellows breath)	
5	Chanting of OM (3 times)	
6	Sudarshan Kriya (the type of cyclical controlled breathing practice)	
4	BREAKFAST	1 hr (morning)
5	SEVA (Selfless Service) includes	1 hr (morning)
6	Guided meditations	3 hr (morning to afternoon)
7	Lunch	1 hr (afternoon)
8	Guided meditations	3 hr (afternoon to evening)
9	Nature walk	1 hr (evening)
10	Practice of wisdom, spiritual music and chanting	2 hr (evening)
11	Dinner (Same food for all participants)	

### 2.4 Procedures for assessment

#### 2.4.1 Anthropometric assessment

The anthropometric assessment included the measurement of an individual's Height (H), Weight (W), Body Mass Index (BMI), Waist circumference (WC), and Hip circumference (HC). All of these were measured as explained below: *Height (Ht in cm or m):* Height was measured with the help of a regular measurement tape. The individuals were asked to stand on the floor without shoes, having equally distributed weight on both legs, joining their ankles, and sliding clamp was slid to the head of the individual and the reading was noted down from the marked point where the sliding clamp strongly presses one's head. *Weight (Wt in Kg):* Body weight was measured with the help of periodically calibrated electronic balance. During the measurement of weight of the body, light clothing was advised and removal of shoes and other articles *Body mass Index (BMI):* The body mass index (BMI) was calculated by the following formula: BMI = body weight (in kg)/height^2^ (in m^2^).

*Waist Circumference (WC in cm):* Individuals were asked to clothe lightly and were asked to stand upright with their feet 25–30 cm apart, having evenly distributed weight. The inch tape for measurement was fitted around the abdominal girth without compressing the soft tissue. The tape was wrapped around the waist after ensuring that the tape measure is parallel to the floor and not twisted. Individuals were asked to take two normal breaths and on the exhale of the second breath, the tape measure was tightened. The measure of the waist to the nearest 0.5 cm (1/4 inch) was taken. *Hip Circumference (HC in cm):* The hip circumference was measured around the pelvis at the point of maximum protrusion of the buttocks.

#### 2.4.2 Assessment spO2 and BP

Oxygen saturation (sPO2) of the participants was measured using Pulse Oximeter to determine the percentage of hemoglobin in the blood saturated with oxygen. Blood Pressure of the participants was measured using Digital Blood Pressure Monitor to obtain the systolic and diastolic components.

#### 2.4.3 Biochemical assessment

About 5 ml of blood from each participant was drawn for the biochemical assessment. Biochemical assessment of cholesterol, triglycerides, HDL, LDL, and VLDL was carried out by a certified diagnostic laboratory using standard diagnostic procedures acceptable for public utility.

#### 2.4.4 Tridosha assessment

The *prakriti (Tridosha)* analysis was done by the scoring of the standard questionnaire filled by the participants. There are separate sets of questions to determine *Vatta, Pitta,* and *Kapha* dominated subjects.

#### 2.4.5 Neurocognitive and psychological assessment

Neuropsychological tests were administered to assess the attention, memory, verbal fluency, executive functioning, and information processing speed.

#### 2.4.6 Six Letter Cancelation Test (SLCT)

This test was used to measure attention, concentration, and visual scanning abilities and visual‐spatial dysfunctions of the participants. SLCT consists of 22 rows *14 columns of randomly arranged alphabets. Six target letters were supposed to be identified among the randomly distributed alphabets in the stipulated timeframe of 90 s (Pradhan, 2013).

#### 2.4.7 Psychological assessment

*State‐Trait Anxiety Inventory (STAI) (Bilingual):* State Anxiety was measured using a sub‐scale of state and trait anxiety inventory (STAI) (Julian, 2011). *The Warwick‐Edinburgh Mental Well‐being Scale (WEMWBS)*: The Warwick‐Edinburgh Mental Well‐being Scale (WEMWBS) was used to assess the mental well‐being (positive mental health) of the participants (Tennant et al., 2007). *Oxford Happiness Questionnaire (OHQ)*: The Oxford Happiness Questionnaire (OHQ) was used to measure the current level of happiness of the participants (Dambrun et al., 2012).

## 3 RESULT

### 3.1 Baseline characteristics of meditators

A total of 48 participants were recruited in the current study. Two participants were excluded due to the non‐availability of blood samples. Analysis was carried out in 46 remaining participants. The Mean (*SD*) age of the participants was 45.87 (10.15). There were 25 males and 21 females in the study. Anthropometric assessments were carried out at the baseline and after the intervention. The baseline characteristics of the subjects are compiled in Table 3.

**Table 3:** Baseline characteristics of the participants

*N*	46
Age *M* (*SD*)	45.87 (10.15)
Male: Female	25:21
V:P:K Dominated	13:22:11
Height (cm)	165.24 (9.724)
Weight (kg)	73.40 (12.44)
BMI (kg/m^2^)	26.85 (3.84)

### 3.2 Effect of SKY‐meditation

**Figure 1:**
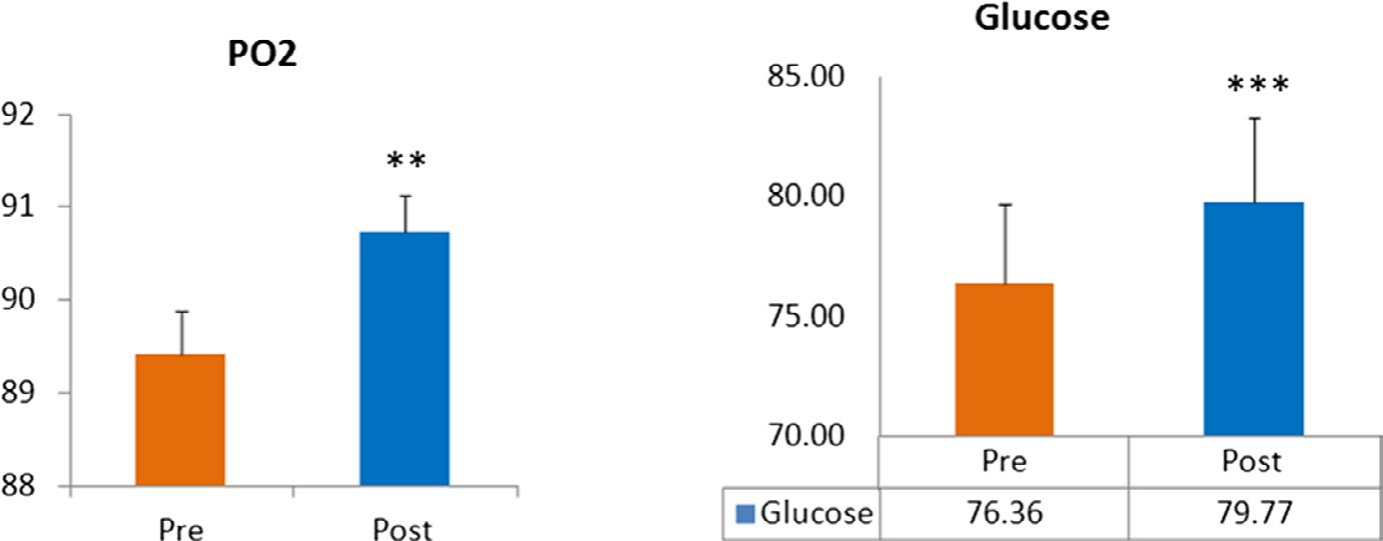
Comparison of PO2 and glucose levels pre‐ and post‐AOL advance meditation (*n* = 46). All results expressed as mean ± *SEM*. Data are statistically analyzed using SPSS 16.0 by the paired *T*‐test. *p < .05, **p < .01, ***p < .001

The comparison of baseline partial oxygen pressure after the SKY‐meditation regimen revealed a significant increase of Po2 (Figure 1). There was a significant enhancement in glucose and reduction in triglycerides and VLDL levels. The estimates of remaining variables have been tabulated (Table 4) along with changes in the biochemical variables. A marked improvement in the anxiety (*t* = 6.89; CI: 4.85–8.85, *p* ≤ .001), Mental well‐being (*t* = 2.49; CI: −4.72 to 0.50, *p* = .017) and happiness index (*t* = −3.58; CI: −14.69 to 4.12, *p* = .001) were obtained.

**Table 4:** Comparison of *anthropometric, Glycemic, Lipid profile, and Neuro‐cognitive factors* pre‐ and post‐AOL advance meditation (*n* = 46)

	*M* ± *SD*	*M* ± *SD*	*t*‐value	CI (95%)		*p* value
				Minimum	Maximum	
Anthropometric						
Weight	73.40 ± 12.45	73.10 ± 11.99	1.02	−0.30	0.92	.314
BMI	26.85 ± 3.85	26.75 ± 3.83	0.83	−0.13	0.32	.410
Waist	95.05 ± 10.08	94.06 ± 10.04	1.35	−0.49	2.47	.185
HIP	103.85 ± 8.38	102.33 ± 12.89	1.12	−1.21	4.25	.268
Systolic	140.47 ± 20.11	137.65 ± 16.62	1.52	−0.91	6.55	.135
Diastolic	94.50 ± 14.05	95.34 ± 16.94	−0.29	−6.60	4.92	.770
PO2	89.33 ± 3.23	90.74 ± 2.65	−2.73	−2.46	−0.37	**.009**
Pulse Rate	86.83 ± 12.40	84.74 ± 11.48	1.07	−1.83	6.00	.289
Glycemic						
Glucose	76.36 ± 22.58	79.77 ± 24.36	−6.75	−4.42	−2.39	**<.001**
Lipid Profile						
Cholesterol	203.91 ± 32.62	200.11 ± 35.76	1.57	−1.08	8.70	.124
Triglycerides	200.49 ± 104.87	194.04 ± 98.51	2.11	0.31	12.59	**.040**
HDL	40.00 ± 4.32	39.91 ± 3.79	0.18	−0.86	1.03	.857
LDL	123.55 ± 24.95	121.60 ± 30.06	0.79	−3.05	6.97	.436
CDL/HDL	5.01 ± 0.89	5.01 ± 0.91	0.07	−0.19	0.20	.943
LDL/HDL	3.04 ± 0.50	3.02 ± 0.73	0.36	−0.12	0.17	.723
VLDL	40.00 ± 21.01	38.64 ± 19.72	2.19	0.11	2.61	**.034**
HB	16.26 ± 1.82	15.99 ± 1.59	3.12	0.10	0.45	**.003**
Stress and Mental Well‐Being						
STAI	35.02 ± 8.05	28.17 ± 7.37	6.89	4.85	8.85	**<.001**
MWB	58.24 ± 7.12	60.85 ± 8.10	−2.49	−4.72	−0.50	**.017**
OHQ	132.40 ± 16.52	141.81 ± 18.23	−3.58	−14.69	−4.12	**.001**
SLCT	28.14 ± 8.65	29.05 ± 8.40	−0.61	−3.89	2.08	.542

Bold represent statistically significant change after advanced meditation protocol.

#### 3.2.1 Effect of change in Po2 level of meditators

**Figure 2:**
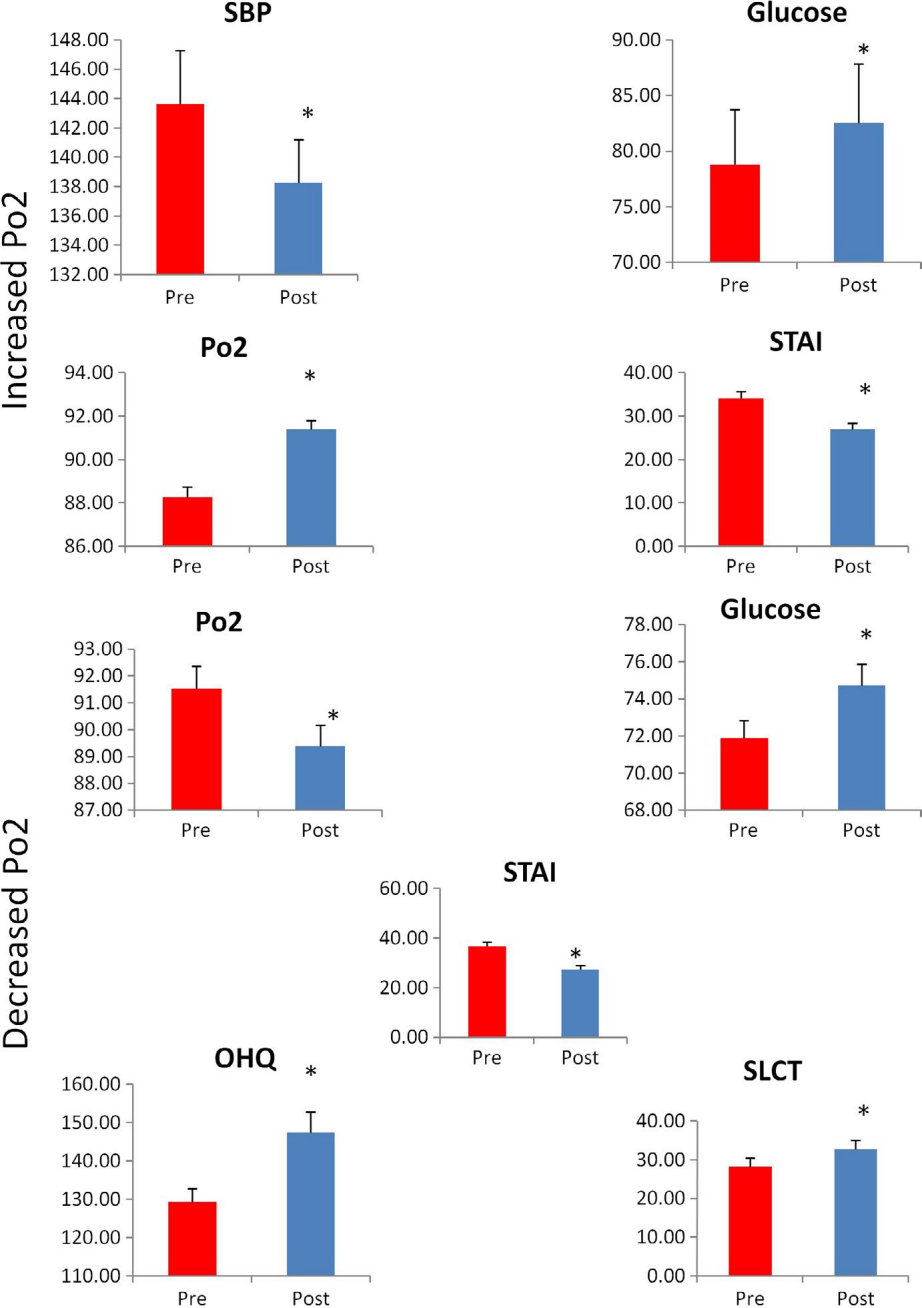
Representing pre‐ and post‐comparison of individuals with decreased (*n* = 15) and increased (*n* = 31) PO2 after AOL advanced meditation. All results expressed as mean ± *SEM*. Data are statistically analyzed using SPSS 16.0 by the paired *T*‐test. *p < .05, **p < .01, ***p < .001

The participants who showed increased Po2 after the SKY were selected in order to analyze the changes in anthropometric, biochemical, lipid profile and psychological indices. Significant enhancement in the levels of Glucose and reduced anxiety were obtained. The Table below shows the changes after SKY. Similarly, subjects with reduced Po2 were determined and the data showed changes in the Glucose and LDL levels as shown in Figure 2. Psychological variables including anxiety and happiness index showed marked changes (Tables 5 and 6).

**Table 5:** Comparison of *anthropometric, Glycemic, Lipid profile, and Neuro‐cognitive factors* pre‐ and post‐AOL advance meditation with increased Po2 (*n* = 31)

Parameters	*M*	*M*	*t*‐value	CI (95%)		*p*‐value
				Minimum	Maximum	
Weight	72.32 ± 11.45	71.68 ± 11.03	1.76	−0.10	1.39	.089
BMI	26.81 ± 3.73	26.58 ± 3.66	1.65	−0.05	0.51	.109
Waist	94.26 ± 9.88	93.23 ± 10.06	1.01	−1.06	3.13	.323
HIP	102.94 ± 8.05	101.00 ± 14.60	0.96	−2.18	6.05	.344
Systolic	143.65 ± 20.22	138.26 ± 16.43	2.56	1.10	9.68	**.016**
Diastolic	96.06 ± 14.27	97.00 ± 19.75	−0.23	−9.36	7.49	.822
PO2	88.26 ± 2.70	91.39 ± 2.26	−6.66	−4.09	−2.17	**<.001**
Pulse Rate	86.00 ± 11.94	83.65 ± 11.24	1.01	−2.43	7.14	.323
Glucose	78.77 ± 27.52	82.52 ± 29.61	−5.90	−5.04	−2.45	**<.001**
Cholesterol	202.23 ± 30.56	199.71 ± 37.01	0.72	−4.64	9.68	.478
Triglycerides	191.97 ± 66.32	185.32 ± 51.74	1.54	−2.15	15.44	.133
HDL	40.26 ± 4.98	40.00 ± 4.13	0.38	−1.11	1.63	.704
LDL	123.35 ± 28.41	122.81 ± 35.68	0.15	−6.91	8.01	.882
CDL/HDL	4.90 ± 0.83	5.00 ± 0.97	−0.57	−0.44	0.25	.572
LDL/HDL	3.00 ± 0.58	3.13 ± 0.92	−0.89	−0.42	0.17	.380
VLDL	38.32 ± 13.30	36.90 ± 10.34	1.62	−0.37	3.21	.117
HB	16.61 ± 1.71	16.23 ± 1.50	3.01	0.12	0.65	**.005**
STAI	34.32 ± 8.40	28.65 ± 7.96	4.60	3.16	8.20	**<.001**
MWB	58.43 ± 7.60	60.63 ± 7.77	−1.84	−4.65	0.25	.076
OHQ	133.03 ± 17.52	138.90 ± 16.85	−1.94	−12.04	0.30	.061
SLCT	28.18 ± 8.96	26.96 ± 7.77	0.63	−2.75	5.18	.535

Bold represent statistically significant change after advanced meditation protocol.

**Table 6:** Comparison of *anthropometric, Glycemic, Lipid profile, and Neuro‐cognitive factors* pre‐ and post‐AOL advance meditation with Decreased Po2 (*n* = 15)

Parameters	*M*	*M*	*t*‐value	CI (95%)		*p* value
				Minimum	Maximum	
Weight	73.93 ± 13.35	74.40 ± 12.30	−0.822	−1.68	0.75	.425
BMI	26.13 ± 3.58	26.40 ± 3.27	−1.740	−0.60	0.06	.104
Waist	94.93 ± 8.47	94.00 ± 7.82	0.989	−1.09	2.96	.339
HIP	104.20 ± 7.17	103.73 ± 7.25	0.507	−1.51	2.44	.620
Systolic	133.60 ± 18.90	136.60 ± 18.07	−0.930	−9.92	3.92	.368
Diastolic	90.60 ± 12.45	91.93 ± 9.40	−0.520	−6.83	4.17	.611
PO2	91.53 ± 3.20	89.402.95	3.702	0.90	3.37	**.002**
Pulse Rate	88.80 ± 13.89	87.20 ± 12.34	0.419	−6.59	9.79	.682
Glucose	71.87 ± 3.76	74.73 ± 4.45	−3.267	−4.75	−0.98	**.006**
Cholesterol	208.73 ± 37.89	202.27 ± 35.09	2.719	1.37	11.57	**.017**
Triglycerides	220.80 ± 161.23	214.20 ± 159.6	1.812	−1.21	14.41	.092
HDL	39.40 ± 2.75	39.60 ± 3.18	−0.408	−1.25	0.85	.689
LDL	124.80 ± 17.41	120.07 ± 14.81	2.424	0.54	8.92	**.029**
CDL/HDL	5.43 ± 1.16	5.00 ± 1.04	3.122	0.13	0.73	**.008**
LDL/HDL	3.29 ± 0.61	3.07 ± 0.47	1.385	−0.12	0.55	.189
VLDL	44.00 ± 32.30	42.67 ± 31.97	1.784	−0.27	2.94	.096
HB	15.73 ± 1.91	15.60 ± 1.76	0.807	−0.22	0.49	.433
STAI	36.47 ± 7.64	27.33 ± 6.41	5.423	5.52	12.75	**.000**
MWB	57.47 ± 6.32	61.00 ± 9.20	−1.610	−8.24	1.17	.130
OHQ	129.33 ± 13.32	147.47 ± 20.70	−3.950	−27.98	−8.29	**.001**
SLCT	28.20 ± 8.	32.73 ± 8.75	−2.105	−9.15	0.09	**.054**

Bold represent statistically significant change after advanced meditation protocol.

#### 3.2.2 Effect of Prakriti Changes in biochemical parameters of meditators

*Prakriti* may contribute to the early acclimatization, subjects with three different *prakrities* were selected for further analysis of different variables. *Vatta* dominated subjects did not show changes in the PO2, anthropometric, and biochemical profile excluding glucose levels. Anxiety and Happiness index were improved. However, PO2 was significantly enhanced in the subjects with *Pitta prakriti*. These subjects also showed a significant reduction in cholesterol and LDL levels. Indicators of psychological variables including anxiety, mental well‐being, and happiness index were also found to have improved after the SKY‐meditation regimen. Similarly, *Kapha* subtypes showed a marked reduction in cholesterol but no psychological variables showed significant changes (Tables 7, 8, 9).

**Table 7:** Comparison of *anthropometric, Glycemic, Lipid profile, and Neuro‐cognitive factors* pre‐ and post‐AOL advance meditation with the domination of *Vatta* Prakiti (*n* = 13)

Parameters	*M* ± *SD*	*M* ± *SD*	*t*	CI (95%)		*p* value
				Minimum	Maximum	
Weight	70.46 ± 9.13	69.92 ± 8.57	1.074	−0.55	1.63	.304
BMI	26.46 ± 3.73	26.31 ± 3.82	0.805	−0.26	0.57	.436
Waist	93.62 ± 7.24	92.31 ± 10.14	0.720	−2.65	5.27	.485
HIP	104.46 ± 8.25	97.92 ± 18.85	1.552	−2.64	15.72	.147
Systolic	137.77 ± 19.02	135.46 ± 12.67	0.593	−6.18	10.79	.564
Diastolic	91.54 ± 15.81	93.46 ± 8.93	−0.376	−13.07	9.23	.714
PO2	88.92 ± 4.11	89.54 ± 2.22	−0.621	−2.77	1.54	.546
Pulse Rate	90.38 ± 15.26	85.69 ± 11.45	1.765	−1.10	10.49	.103
Glucose	76.08 ± 13.46	79.77 ± 14.01	−3.322	−6.11	−1.27	**.006**
Cholesterol	189.62 ± 25.28	194.62 ± 47.72	−0.677	−21.10	11.10	.512
Triglycerides	188.62 ± 58.87	176.23 ± 41.61	1.605	−4.43	29.20	.135
HDL	37.46 ± 5.03	39.08 ± 2.99	−1.790	−3.58	0.35	.099
LDL	113.08 ± 24.47	120.38 ± 46.70	−0.966	−23.79	9.17	.353
CDL/HDL	5.00 ± 0.74	5.00 ± 1.35	0.000	−0.77	0.77	1.000
LDL/HDL	2.92 ± 0.67	3.33 ± 1.23	−1.449	−1.05	0.22	.175
VLDL	37.77 ± 11.87	35.15 ± 8.41	1.673	−0.79	6.02	.120
HB	15.69 ± 2.14	15.54 ± 1.90	1.000	−0.18	0.49	.337
STAI	36.77 ± 8.36	29.46 ± 6.78	3.611	2.90	11.72	**.004**
MWB	57.77 ± 6.64	62.46 ± 6.09	−2.145	−9.46	0.07	.053
OHQ	131.85 ± 18.01	148.92 ± 17.84	−2.522	−31.83	−2.32	**.027**
SLCT	29.64 ± 7.88	31.73 ± 10.62	−0.623	−9.57	5.39	.548

Bold represent statistically significant change after advanced meditation protocol.

**Table 8:** Comparison of *anthropometric, Glycemic, Lipid profile, and Neuro‐cognitive factors* pre‐ and post‐AOL advance meditation with the domination of *Pitta Prakriti (n = 22)*

Parameters	*M* ± *SD*	*M* ± *SD*	*t*	CI (95%)		*p* value
				Minimum	Maximum	
Weight	72.82 ± 10.39	72.73 ± 10.15	0.200	−0.86	1.04	.844
BMI	26.55 ± 2.94	26.55 ± 2.84	0.000	−0.31	0.31	1.000
Waist	94.95 ± 7.05	95.02 ± 6.38	−0.081	−1.81	1.67	.936
HIP	103.73 ± 6.25	103.41 ± 8.52	0.317	−1.77	2.40	.754
Systolic	145.14 ± 18.79	142.23 ± 19.49	1.268	−1.86	7.68	.219
Diastolic	98.09 ± 11.65	98.64 ± 22.89	−0.109	−10.97	9.88	.914
PO2	89.77 ± 1.93	91.59 ± 2.17	−3.058	−3.05	−0.58	**.006**
Pulse Rate	84.91 ± 11.05	84.77 ± 11.86	0.050	−5.54	5.81	.961
Glucose	79.45 ± 31.35	83.14 ± 33.78	−4.978	−5.22	−2.14	**.000**
Cholesterol	212.68 ± 36.68	205.27 ± 34.99	3.711	3.26	11.56	**.001**
Triglycerides	224.32 ± 138.56	218.55 ± 134.12	1.385	−2.90	14.44	.181
HDL	40.36 ± 4.23	39.14 ± 4.53	1.618	−0.35	2.80	.121
LDL	127.64 ± 26.51	122.68 ± 24.07	2.271	0.42	9.49	**.034**
CDL/HDL	5.18 ± 1.18	5.18 ± 0.91	0.000	−0.34	0.34	1.000
LDL/HDL	3.18 ± 0.59	3.09 ± 0.61	0.624	−0.21	0.39	.540
VLDL	44.68 ± 27.75	43.50 ± 26.88	1.404	−0.57	2.93	.175
HB	16.91 ± 1.60	16.45 ± 1.57	2.887	0.13	0.78	**.009**
STAI	35.23 ± 6.81	26.59 ± 4.74	6.341	5.80	11.47	**.000**
MWB	58.43 ± 7.18	61.10 ± 7.27	−2.086	−5.33	0.00	**.050**
OHQ	133.95 ± 14.53	140.23 ± 16.78	−2.185	−12.24	−0.30	**.040**
SLCT	28.10 ± 9.71	29.38 ± 7.26	−0.604	−5.73	3.15	.553

Bold represent statistically significant change after advanced meditation protocol.

**Table 9:** Comparison of *anthropometric, Glycemic, Lipid profile, and Neuro‐cognitive factors* pre‐ and post‐AOL advance meditation with the domination of Kapha Prakriti (*n* = 11)

Parameter	*M* ± *SD*	*M* ± *SD*	*t*	CI (95%)		*p* value
				Minimum	Maximum	
Weight	79.64 ± 17.35	79.00 ± 16.88	0.939	−0.874	2.147	.370
BMI	28.09 ± 5.68	28.00 ± 5.55	0.319	−0.543	0.725	.756
Waist	98.55 ± 15.98	95.77 ± 14.72	1.777	−0.703	6.249	.106
HIP	104.09 ± 12.34	105.82 ± 11.91	−1.136	−5.114	1.660	.282
Systolic	138.00 ± 23.13	133.50 ± 11.76	0.933	−6.244	15.244	.373
Diastolic	93.18 ± 16.04	92.55 ± 7.54	0.151	−8.737	10.009	.883
PO2	88.90 ± 4.48	90.00 ± 3.30	−0.729	−4.513	2.313	.485
Pulse Rate	87.73 ± 11.24	83.45 ± 12.27	0.795	−7.696	16.242	.445
Glucose	71.27 ± 2.87	74.00 ± 4.86	−3.012	−4.745	−0.710	**.013**
Cholesterol	205.64 ± 28.25	198.09 ± 20.85	2.591	1.057	14.034	**.027**
Triglycerides	171.73 ± 59.05	170.09 ± 47.22	0.385	−7.828	11.100	.708
HDL	42.18 ± 1.99	42.45 ± 1.69	−0.820	−1.014	0.469	.432
LDL	129.18 ± 20.76	121.91 ± 17.44	3.043	1.948	12.597	**.012**
CDL/HDL	4.91 ± 0.70	4.55 ± 0.52	2.390	0.025	0.703	**.038**
LDL/HDL	3.09 ± 0.54	2.91 ± 0.54	1.491	−0.090	0.454	.167
VLDL	34.27 ± 11.86	33.82 ± 9.28	0.495	−1.591	2.500	.631
HB	16.18 ± 1.17	15.91 ± 0.94	1.399	−0.162	0.707	.192
STAI	32.36 ± 10.23	29.27 ± 11.62	1.623	−1.152	7.334	.136
MWB	58.73 ± 8.38	58.73 ± 11.67	0.000	−5.669	5.669	1.000
OHQ	131.91 ± 19.27	138.64 ± 20.51	−1.315	−18.125	4.670	.218
SLCT	25.64 ± 6.90	25.73 ± 8.06	−0.033	−6.222	6.040	.974

Bold represent statistically significant change after advanced meditation protocol.

## 4 DISCUSSION

Acclimatization at high altitude requires a synergistic effect of various body systems based on individual genetic, epigenetic, and Psychometric (*Prakriti*) constitution (Brown & Rupert, 2014). The understanding of the varying effects of high altitude sickness in different individuals is limited. Moreover, current treatment modalities works symptomatically along with side effects which may be prevented by yoga‐based interventions. Hence, our study provides an introduction to the effect of SKY‐based advance meditation at high altitude.

Interesting results were obtained when genetic variants were studied in the Sherpa communities in which EPAS1 Single Nucleotide variations (rs13419896, rs4953354 and rs4953388) were reported to be contributory (Hanaoka et al., 2012). These SNPs provide biochemical protection against oxidative damage in high altitude conditions (Horscroft et al., 2017) and also highlight the importance of examining *Prakriti* wise biochemical profile when Yoga or other related interventions are carried out in tandem with the conventional medical assessment. This indicates a community wise adaptation to the high altitude conditions that can also be examined in meditators. In this context, our study also showed positive effects of the SKY‐meditation regimen in the high altitude area (Leh). An increase in glucose has been noticed ascribed to homeostatic changes in glycolysis in meditators. Certain studies have reported enhanced ATP requirements fulfilled by increased glycolytic enzymes in the hypoxic conditions (Horscroft et al., 2017). Our study is consistent with this finding. Under normal circumstances, the baseline glucose levels after the ascent to the high altitude. Whether performing of SKY can result in enhanced the cellular availability of oxygen concomitant with homeostatic upregulation of glucose levels can only be analyzed by additional molecular investigations.

An increase in the percent oxygen saturation among meditators indicates a crucial role of SKY led acclimatization regime. Furthermore, individual variations in the oxygen saturation indicate that personalized variations may enhance the susceptibility to acclimatization process and therefore, *Prakriti* or *Ayurgenomic* analysis can provide vital clues in this analysis. Independent mechanisms may govern the increase or decrease in the Po2 even if a similar Yoga module is followed. Hence, comparative studies with various Yoga modules at various heights may be useful in developing a deeper understanding of the molecular and genetic aspects of mind‐body techniques. This phenomenon results in two different molecular signaling pathways. Reduction in Po2 results in the upregulation of hypoxia inducing factors (HIFs) which further activates several metabolic cascade events including the depletion of cholesterol (Lee et al., 2009). Similarly, the Po2 reduction in Yoga performing subjects shows the depletion of cholesterol levels. Similar depletion was observed in the subjects with *pitta* and *kapha* prakriti types but not in the *Vatta* group.

SKY seems to induce the improvement in the level of anxiety, stress, and mental well‐being under high altitude conditions. However, subjects with increased Po2 only demonstrated improvement in anxiety in contrast to the subjects with decreased Po2 who along with improved anxiety, showed improvements in the happiness index. Probably, improvements from the more hypoxic conditions results in improved cognitive performance. The selection of the Himalayan region for obtaining enlightenment in ancient India provides a clue to enhanced cognitive functioning and psychological benefits when coupled with Yogic regime under hypoxic conditions.

Our study indicates the role of *prakriti* in driving biochemically mediated acclimatization at high altitude. However, genetic and epigenetic associations for undergoing mechanisms need to be established in further studies. Larger study with additional variables and molecular analysis can provide the preventive strategies important for the analysis of personality traits or *Prakriti*. For instance, the subjects with “*pitta prakriti”* responded better in the adverse hypoxic high altitude conditions than the other types. Although, SKY‐Meditation regimen improved overall health during acclimatization, the decrease in percent oxygen saturation, and related health benefit requires further analysis.

## CONFLICT OF INTEREST

The authors declare that they have no competing interests.

## AUTHOR CONTRIBUTIONS

AA: Conceptualisation of the manuscript. DB: Conceptualization of the study, regulatory approvals, logistic support, Supervision pertaining to Collection of data, final approval. NL: Conceptualization of the study, Conduct of SKY intervention, Supervision pertaining to Collection of data, Manuscript drafting and final approval. RT: Analysis and interpretation of data, preparation, and editing of the manuscript and its submission.

## ETHICAL APPROVAL

The study was approved by the Institutional ethics committee of SSIAR, Bangalore (vide SSIAR/IEC/05) as well as PGIMER, Chandigarh (PGI/IEC/2019/000643). All participants provided consent for the participation under the guidelines of IECs.

## Data Availability

The datasets used and/or analyzed during the current study are available from the corresponding author on reasonable request.
